# Stress-free Everyday LiFe for Children and Adolescents REsearch (SELFCARE): a protocol for a cluster randomised trial testing a school teacher training programme to teach mindfulness (“.b”)

**DOI:** 10.1186/s40359-021-00530-9

**Published:** 2021-02-17

**Authors:** Lise Juul, Morten Frydenberg, Michelle Sand Beck, Lone Overby Fjorback

**Affiliations:** 1 Department of Clinical Medicine, Danish Center for Mindfulness, Aarhus University, Gudrunsvej 78, 3, 8220 Brabrand - Aarhus, Denmark; 2 MFStat, Aarhus, Høgemosevej 19A, 8380 Trige, Denmark

**Keywords:** Pragmatic clinical trial (MeSH), Community mental health services (MeSH), Stress, Psychological (MeSH), Schoolbased intervention (MeSH), Mindfulness-based stress reduction, MBSR, Mindfulness, Effectiveness

## Abstract

**Background:**

There is a call for sustainable, evidence-based interventions in schools to promote mental health in schoolchildren. Our primary aim of this trial is to evaluate the effectiveness in vulnerable pupils of a school teacher training programme to teach mindfulness (“.b” programme) as a part of compulsory class room teaching in Danish schools on the pupils’ self-reported mental health at 6-month follow-up. Our secondary aim is to evaluate the effectiveness of the school teacher training programme to teach the “.b”-programme as a part of compulsory class room teaching among the total pupil population on the pupils’ self-reported mental health at 3 and 6 months after baseline.

**Methods:**

The pragmatic cluster two-armed randomised controlled trial includes 110 municipal or private schools from all five regions in Denmark; 191 school teachers and approximately 2000 pupils at 11–15 years of age. Exclusion criteria; for schools: < 100 pupils; for pupils: parental opt out. Our intervention consists of (A) a school teacher training programme and (B) the “.b”-programme delivered as part of compulsory class room teaching in schools to pupils at the age of 11–15 years. The pupils in the control schools receive education as usual. Our primary study population is the vulnerable subgroup with a Strengths and Difficulties Questionnaire (SDQ) total difficulties score > 80% percentile at baseline (approximately 400 pupils). The primary outcome is change in the SDQ total score by the pupils. We also evaluate the effectiveness among the total pupil study population and in girls and boys, respectively and use other measures on mental health. Data will be analysed with repeated measurement models taken clusters into account.

**Discussion:**

This large-scale trial will estimate the effectiveness of a population-based strategy on mental health in Danish schoolchildren. The trial evaluates the effect of a school teacher training programme, where teachers are trained in teaching the “.b” programme. The “.b” programme will be taught as a part of compulsory class room teaching. The intervention takes implementation issues into account. Effectiveness will be evaluated both in a vulnerable subgroup and among the total population.

*Trial registration number *ClinicalTrials.gov Identifier: NCT04208113, registered December 23 2019, https://clinicaltrials.gov/ct2/show/NCT04208113.

## Background

The World Health Organization (WHO) defines mental health as “a state of well-being in which every individual can realize his or her own potential, can cope with the normal stresses of life, can work productively and fruitfully, and is able to make a contribution to his or her community” [1]. Regarding children, WHO elaborates that “an emphasis is placed on the developmental aspects, for instance, having a positive sense of identity, the ability to manage thoughts, emotions, as well as to build social relationships, and the aptitude to learn and to acquire an education, ultimately enabling their full active participation in society” [1]. Hence, mental health includes both well-being and functioning. Sound mental health is a public good. Fortunately, the majority of children in Denmark as well as world-wide are in good health [2, 3]. However, depression is a leading cause of disability worldwide and suicide is the second leading cause of death in the group of 15–29-year-olds [4].

Mental health is much more than just the absence of a mental disorder. Mental health problems can be seen along a continuum from mild, time-limited distress to chronic, progressive, and severely disabling conditions [5]. Importantly, mental health can be improved along the entire continuum to avoid problems associated with suffering, functional impairment, stigma and other concerns such as educational achievements, substance use and abuse, self-harm and violence [2]. Since 2002, the Danish part of the international research project Health Behaviour in School-aged Children has reported a decrease in the proportion of 15-year-olds reporting a high life satisfaction [3]. The latest survey showed increases in irritability and nervousness among Danish school-aged children [3], and the recent National health profile showed a significant decrease in the mental health among the 16–24-year-olds [6]. A high occurrence of stress, anxiety, tension and loneliness was seen in this age group [6]. Adolescence is the period of life when most mental disorders surface. In 50% of adults with a mental disorder, the first symptoms occurred before the age of 15 years [7]. Skovlund et al. in their register-based study showed an increase in depression diagnoses and prescribed antidepressants among Danish young girls between 2000 and 2013 [8].

In addition to human suffering, mental health problems have major socio-economic consequences. In 2015, the total costs of mental health illness in Europe were estimated to be more than EUR 600 billion. Among the European countries, Denmark was the country with the highest costs in terms of % of the gross domestic product (5.38%) [9].

Stress and adversity are parts of life and there are appropriate ways to deal with this [10]. Therefore, it would be desirable to provide school-aged children with competencies to promote mental health and thus prevent mental health problems. In The Lancet Commission on Global Mental Health and Sustainable Development, Patel et al. stressed the importance of improving mental health for whole populations [5]. This is in line with G. Rose suggesting that shifting the whole curve in a favourable direction has the highest and most valuable impact at a society level [11]. The population-based strategy e.g., using universal interventions has the potential to improve mental health in high risk and vulnerable groups without causing stigma. Simultaneously, it also has the potential to improve resources in all children and to prevent children from being at high risk for mental health problems [11]. O’Connor et al. concluded that the school environment is a highly relevant setting for the provision of mental health promotion [12]. However, robust evidence to guide intervention content is lacking [12, 13]. Dunning et al. concluded positive effects of mindfulness-based intervention (MBI) on mental health in children and adolescents [14]. Mindfulness can be defined as “the awareness arising through paying attention on purpose in the present moment, non-judgmentally, in the service of self-understanding, wisdom, and compassion” [15]. Schoolchildren learn to pay attention by using short mindfulness practices. They practice becoming familiar with sensations in the body, and their thoughts and feelings associated with pleasant and unpleasant experiences. Increased awareness and embodied presence and knowledge have the potential to enhance the regulation of emotions and behaviour, well-being and social competences [16]. The meta-analysis by Dunning et al. was based on 33 randomised controlled trials with 3666 children and adolescents. However, there was a high degree of variability in intervention contents and target groups [14]. The “.b” programme was the most frequently MBI programme applied in school settings [12, 14]. The “.b” programme, is a curriculum-based classroom introduction to mindfulness, which has been systematically developed and tested during the past 10 years in England [16, 17]. The content is based on the evidence-based mindfulness programmes for adults, Mindfulness-Based Stress Reduction (MBSR) [10, 18] and Mindfulness-Based Cognitive Therapy (MBCT) [19]. However, the duration of the sessions and the mindfulness practices are shorter, and there is less enquiry. The content of MBSR/CT is adapted to appeal to teenagers and work in a mainstream classroom setting. It has been developed and adapted to ensure that it is acceptable within diverse school contexts and student populations. “.b” is an universal intervention in that it is intended to be taught to all pupils in regular school class rooms and not to selected groups of pupils. The curriculum says “At the most basic level, “.b” aims simply to be an awareness raising exercise that gives all students a taste of mindfulness, so that they know about it, and can thus return to it later in life, learning more about it when this is useful to them”. Moreover, the “.b” programme has also been shown to have a potential effect on mental health such as symptoms of depression [17]. Hence, the “.b” programme has the potential to shift the whole curve concerning mental well-being in a favourable direction [16]. The proportion of schoolchildren with good mental health and thus less room for improvement will always dilute effects in total populations [11]. One will therefore expect to find small effect sizes in the total population of schoolchildren and higher effect sizes in a vulnerable subgroup. This hypothesis has been supported by a pilot study that was conducted by our research group.

Large scale trials in the UK and Finland evaluated the effectiveness of the “.b” programme [16, 20]. The Finnish trial by Volanen et al. recently showed effectiveness of “.b” compared to an active control intervention on resilience and behavioural and emotional functioning [21]. The effects were solely seen among girls [21]. The “.b” programme teaching in the Finnish study was provided by professional mindfulness teachers [21]. Hence, this trial did not evaluate the effectiveness of a school teacher training programme to teach the “.b” programme. More recently, there has been a shift away from using specialist staff to implement interventions in real-life circumstances, and instead using those routinely involved in the life of the school, such as teachers. The “.b” programme is developed by and to be used by school teachers. Weare et al. suggested this approach in their review in order to achieve long-term sustainability [22]. This leaves a knowledge gap on how to optimally train school teachers to skilfully teach such interventions while minimising the potential for harm [23]. The ambition for high quality mindfulness education is to ensure mindfully embodied presence along with appropriate skills and frameworks that support human growth and flourishing [23]. Implementation research has shown great challenges in training school teachers to implement and to become competent to teach the “.b”-programme in English schools [24]. The authors suggests adding support to the school teachers when they start teaching “.b” in their respective schools, to the existing teacher training route. The existing teacher training route in the UK, was comprised of an 8-session instructor-led personal mindfulness course, combined with a 4-day training on how to teach the “.b”-programme [24]. The quality of the training of school teachers to teach mindfulness in schools may therefore be crucial and implementation issues must be taken into account. O’Connor highlights the importance of providing school teachers with the necessary skills and knowledge to ensure that the school setting continues to be a beneficial environment for promoting mental health [12]. Results of implementation research have also shown that beyond high quality teacher training, engaged teachers and supportive head teachers are important for implementation [25]. The Danish Parliament has funded the Danish Center for Mindfulness at Aarhus University to educate school teachers to teach mindfulness to pupils at lower secondary education levels across Denmark. We have developed a school teacher training programme that take the above-mentioned issues into account.

## Aims

Our primary aim of this trial is to evaluate the effectiveness among vulnerable schoolchildren of a school teacher training programme to teach mindfulness (“.b”) as a part of compulsory class room teaching in Danish schools on the pupils’ self-reported mental health at 6-month follow-up.

Our secondary aim is to evaluate the effectiveness of the school teacher training programme to teach the “.b” programme as a part of compulsory class room teaching among the total population of schoolchildren on their self-reported mental health at 3 and 6 months after baseline.

## Methods/design

### Trial design

We designed the trial as a pragmatic cluster randomised two-armed trial.

### Study setting

In Denmark, compulsory education is 10 years (0–9). The primary (classes 0–4) and lower secondary (classes 5–9) education covering the Danish public-school programme The Folkeskole. Currently, there are 1339 (71%) municipal schools and 538 (29%) private schools in Denmark [26]. In October 2018, it was estimated that 17.7% of the Danish children and adolescents (classes 0–9) went to a private school [27].

### Eligibility criteria

In this trial, we included municipal and private schools from all of the five regions in Denmark with > 100 pupils. We excluded schools with < 100 pupils because of the lack of sufficient lower secondary school classes for the teacher to teach in the project. Consent to school participation was given by school headmasters. We allowed each school to include 1–3 teachers teaching pupils at lower secondary education for teacher training. Inclusion criteria for the pupils were age of 11–15 years and thus pupils in lower secondary school (classes 5–8). The trial exclusion criterion was parental opt out on behalf of their children not to complete the trial questionnaires.

### Intervention

**Fig. 1:**
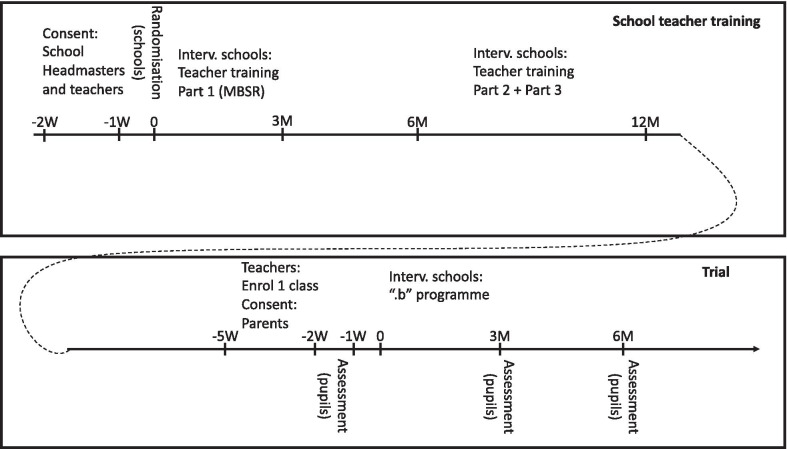
A brief overview of the SELFCARE project

**Fig. 2:**
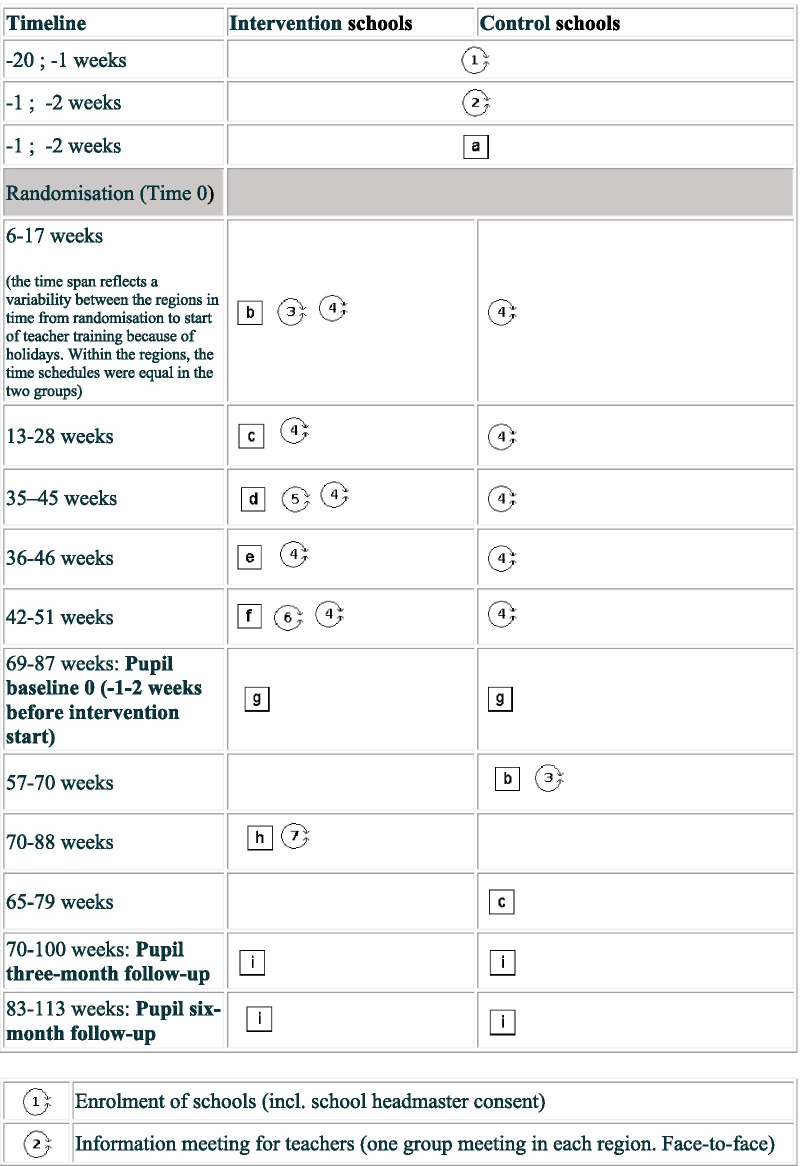

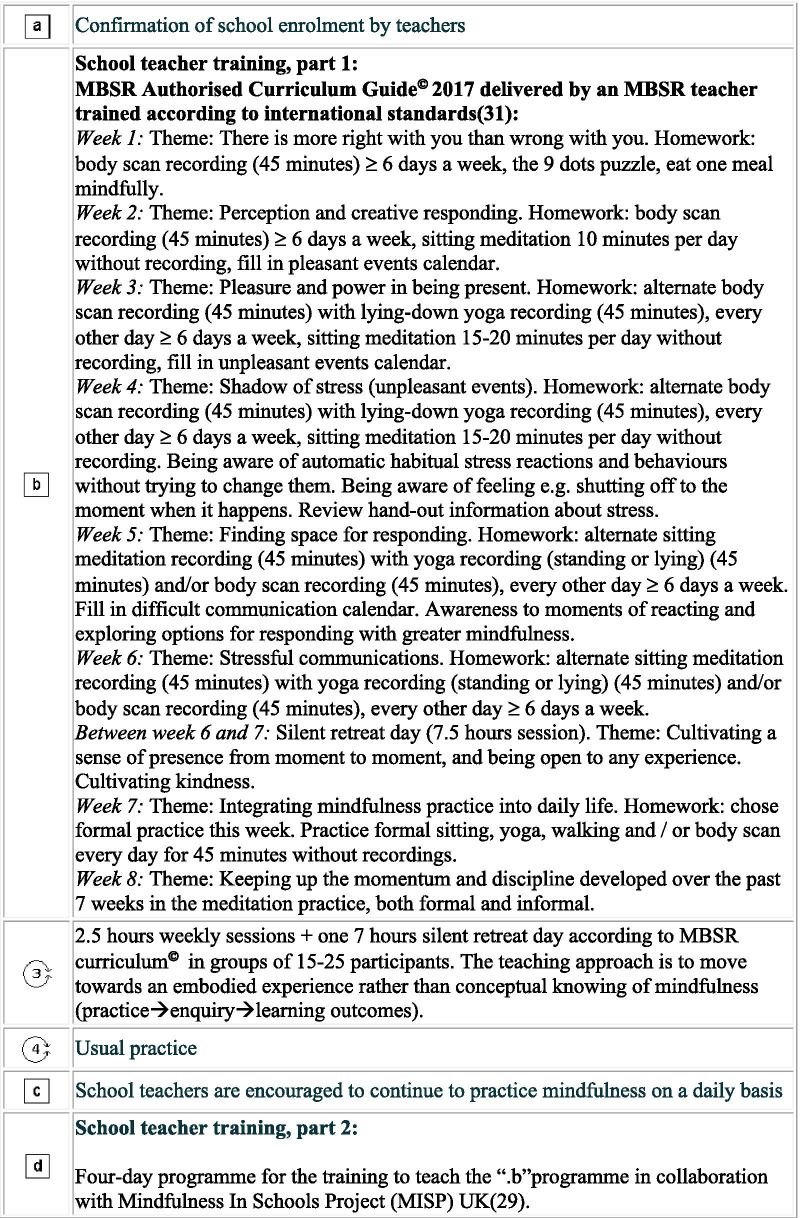

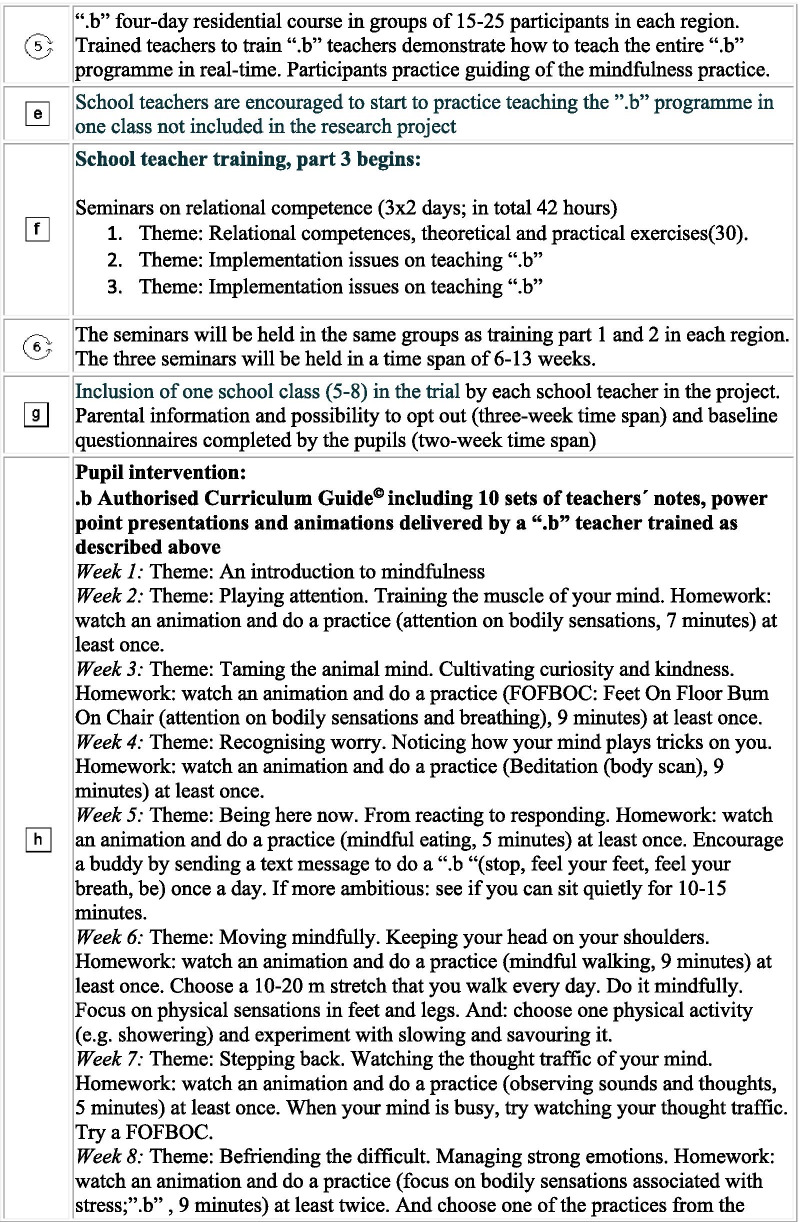

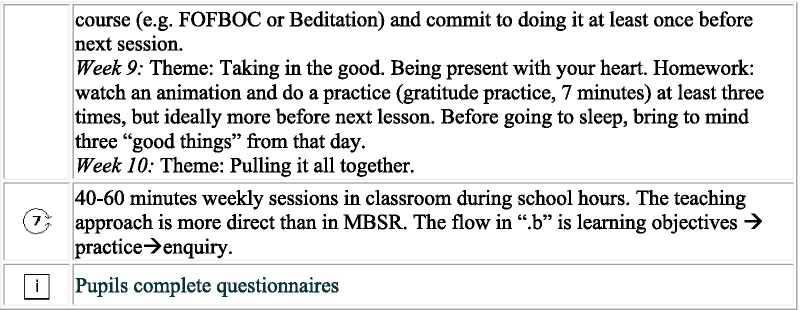
Timeline and description of research activities and intervention contents in the SELFCARE project. A cluster randomised trial testing a school teacher training programme to deliver the “.b” programme in 110 schools from all five regions in Denmark. (Squares reflect fixed elements and circles reflect that some factors e.g. relational dynamics in groups are never fixed.)

Our intervention is a multi-level, multi-component complex intervention. It consists of (A) a school teacher training programme and (B) the “.b” programme to be delivered to pupils at the age of 11–15 years as a part of compulsory class room teaching. The teacher training is being conducted during a period of approximately one year from randomisation. The schools were randomised to begin teacher training in 2019 (intervention group) or in 2020 (control group) in blocks covering the five Danish regions: December 2018: Central Denmark Region; March 2019: The Capital Region of Denmark and Region Zealand; May 2019: Region of Southern Denmark and North Denmark Region. A brief overview of the trial is outlined in Fig. 1. In more detail, the timeline for research activities and intervention content for the trial is depicted in Fig. 2 in a PATPlot according to Perera et al. [28].

Two MBSR instructors from Danish Center for Mindfulness, Aarhus University (one with a professional background as a psychologist and one as an upper secondary school teacher) were trained by the Mindfulness In Schools Project (MISP) in the UK [29] to teach school teachers to teach the “.b” programme.

Our school teacher training programme includes three parts: (1) the establishment of a mindfulness practice by participation in the MBSR programme and sustaining the mindfulness practice by formal daily practice; (2) completion of the four-day “.b” residential course [29], and (3) completion of the three two-day seminars on relational competencies and implementation issues regarding teaching the “.b” programme based on the work of The Danish Association for Promoting Life Wisdom in Children”[30].

The “.b” programme consists of well-described, weekly 40–60-min classroom sessions over 10 weeks [29]. All sessions have a specific theme, teachers’ notes, power point presentations and animations. All materials have been translated into Danish in collaboration with MISP.

The teachers in the control group begin teacher training in 2020/21, but we ensure that they do not begin part 2 (the training to teach “.b” and the access to the teaching material) before we have collected data from the 6-month follow up among the pupils.

### Outcomes

The outcome measures are described in Table 1.

**Table 1:** Description of outcome measures, assessed at baseline, 3 and 6 months among schoolchildren, the SELFCARE project

Measurement	Description
Demographic data	Data on age, sex, school, class, cohabitation and family socio-economic status will be collected as in the national Health Behaviour in School-aged Children survey
The Strengths and Difficulties Questionnaire (SDQ)-youth self-report [32]	The SDQ comprises 25 items describing psychological or behavioural attributes. The items have three response categories ‘‘very true’’, ‘‘somewhat true’’ or ‘‘not true’’. The instrument can generate five sub-scales scores (emotional problems, conduct problems, hyperactivity, peer problems, and prosocial behaviour). The total difficulties score is the sum of the subscale scores excluding the score of prosocial behaviour. The total difficulties score ranges from 0 to 40 with higher values indicating poorer behavioural and emotional functioning and well-being. The subscales ranges from 0 to 10 with higher scores indicating poorer functioning and well-being for four of the subscales (emotional, conduct, hyperactivity–inattention and peer problems) and better functioning and well-being for one of the subscales (prosocial). Goodmann et al. has shown a dose–response relationship between total difficulties scores and the risk of having or in a 3-year period developing a mental disorder [33]. The odds-ratio for having a mental disorder per one-point increase in the youth self-reported SDQ total difficulties score was 1.23 (95%CI 1.21 to 1.25). The odds-ratio for developing a mental disorder with-in a 3-year period per one-point increase in the youth self-reported SDQ total difficulties score was 1.16 (95% CI 1.13 to 1.18). These are results of a population-based observational study. However, Goodmann et al. argues that differences in mean total difficulties scores between intervention and control groups in experimental research can legitimately be interpreted as reflecting genuine differences in mental health [33]
Warwick-Edinburgh Mental Wellbeing Scale (WEMWBS) -short version [37–39]	The seven-item WEMWBS scale is designed to capture a broad conception of well-being covering both hedonic (happiness, subjective wellbeing) and eudaemonic (positive functioning) wellbeing in general populations [37]. The items are all positively worded and have five response categories “none of the time”, “rarely”, “some of the time”,” often”, “all of the time”. The score is a sum-score with the range of 7–35 with higher scores indicating higher levels of well-being. The raw score will be converted to a metric score as recommended. The scale has shown properties of having no ceiling effect and having sensitivity to detect changes [37]. WEMWBS has been validated for use in adults from the age of 16 years in e.g. the UK [37] and Denmark [38]. It has also been found appropriate for use in adolescents from the age of 13 in England and Scotland [39]. Kuyken et al. showed effect on WEMWBS of.b in a pilot study [17]*.* WEMWBS is included in the latest Danish contribution [3] of “The Health Behaviour in School-aged Children (HBSC) – a World Health Organization Collaborative Cross-national Study”
Brief Resilience Scale (BRS) [40]	The BRS is a six-item measure of resilience [40]. Item responses range from 1–5. A summary score is created that averages across the six items (range = 1–5), with higher scores indicating a greater ability to bounce back when experiencing adversity [40]. The following cut-off points have been suggested: Scores from 1.00–2.99: low resilience; 3.00–4.30: normal resilience; 4.31–5.00: high resilience [40]. Windle G et al. has proposed BRS to be one of the most valid instruments to measure resilience in their review of psychometric rigor of resilience measurement scales [41]. However, the validation has only been conducted in adult populations
School connectedness and bullying items from the Danish student well-being questionnaire (DSWQ) [42]	Since 2014, The Danish Ministry of Education has monitored well-being among all Danish public-school students on a yearly basis by use of a self-developed 40-item questionnaire. From these items, Niclasen et al. has proposed a four-factor structure based on factor analysis [42]. We will use the seven-item school connectedness scale with items such as “I feel that I belong at this school” and “Most of the students in my class are kind and helpful” as outcome in our trial. Furthermore, we will use two bullying items from DSWQ as recommended by Niclasen et al. [42]
EQ-5D-Y [43]	EQ-5D is a valid instrument to measure health-related quality of life (HRQoL) for use in economic evaluations. A child-friendly generic EQ-5D-Y has been developed and has shown to be feasible in assessing HRQoL in the age of 8–15 years [43]. It comprises five dimensions: mobility (“walking about”), self-care (“looking after myself”), usual activities (“doing usual activities”), pain or discomfort (“having pain or discomfort”), and anxiety or depression (“feeling worried, sad or unhappy”). Furthermore, respondents are asked to rate their overall health on the EQ VAS, a vertical scale from 0, labelled as “The worst health you can imagine” to 100, labelled as “The best health you can imagine”. The EuroQol Research Foundation is currently working on the development of a value set in the EQ-5D-Y context, which will make cost effectiveness analysis feasible [43]
Sleep quality [44]	A scale consisting of seven close-ended questions with three ordinal response categories ranked from 1 to 3 will measure the quality of sleep. Minimum score is 7 (sleeping badly) and maximum score 21 (sleeping well) [44]
Child-Adolescent Mindfulness Measure (CAMM) [45]	The 10-item CAMM measure mindfulness. It has been developed and validated among children and adolescents 10–17 years. Lower scores indicate higher levels of mindfulness [45]
Mindfulness practice (those allocated to.b) [17]	We will use questions on adherence that have been used in former.b research [17]

#### Primary outcome

We use self-reported changes from baseline in the SDQ total difficulties score [32] by the pupils as the primary outcome. SDQ measures behavioural and emotional functioning and social competencies, which is highly relevant according to WHO´s definition of mental health e.g. the ability to manage thoughts, emotions, as well as to build social relationships [1]. SDQ is recommended as a measure of child well- being in community settings such as schools [33].

Our primary end-point is 6 months after baseline. Our primary study population is the vulnerable sub group with a SDQ total difficulties score above the 80th percentile [34]. They will be taught the “.b” programme together with the whole class as a part of compulsory class room teaching.

#### Secondary outcomes

Changes from baseline in all other outcomes described in Table 1 are secondary outcomes. We evaluate the effectiveness in a vulnerable sub group, in boys, in girls and among the total pupil population.

#### Implementation

Reasons for teachers discontinuing the trial will be registered. We also register teacher participation in the training programme. The following is required for the school teachers to acquire the “b”-teaching certificate: (1) completing at least six out of nine MBSR sessions, (2) a four-day residential course, and (3) at least two out of three seminars on relational competences and implementation issues on teaching “.b”. Furthermore, we will register the number of sessions of the “.b” programme that school teachers teach in the classes they enrolled in the trial. The school teacher training programme includes information on the potential adverse effects associated with mindfulness [35, 36]. The school teachers will be encouraged to report all observed adverse events.

### Sample size

Based on our pilot study, we expect a reduction of 1.5 score points (sd 3.8) in the SDQ total difficulties score among the vulnerable sub group. To detect this effect with a statistical power of 80%, it requires a study sample of 102 pupils in each group; 204 in total.

To detect a reduction of 0.7 score points (sd 5.5) in the SDQ-total difficulties score among the total pupil population, requires a study sample of 971 pupils in each group; 1942 in total.

### Recruitment

From May 2018—May 2019, we recruited schools through letters to school headmasters, local information meetings and by advertising on our website (www.mindfulness.au.dk) and other social media (Facebook and Instagram). We informed the school headmasters and interested teachers that the schools would be randomised to begin teacher training in 2019 (intervention group) or in 2020 (control group). We further informed that it was an obligation for each teacher to recruit one 5–8 class with 15 to 28 pupils in a year from randomisation corresponding to the time of the completion of teacher training in the intervention group to test the effectiveness of the “.b”-programme. We recruited schools in five blocks defined by the five regions. We have included a total of 110 Danish schools and 191 teachers who consented to participate in the trial; 27 schools and 56 teachers from the Capital Region of Denmark, 18 schools and 28 teachers from Region Zealand, 25 schools and 42 teachers from Region of Southern Denmark, 30 schools and 50 teachers from Central Denmark Region and 10 schools and 15 teachers from North Denmark Region.

We expect teachers to recruit approx. 2000 pupils in total for the trial. We expect that approx. 400 pupils will be in the vulnerable sub group resulting in a SDQ total difficulties score > 80% percentile at baseline.

In this real-life setting, it was not possible to include the pupils before the randomisation of the schools due to the logistics of teacher planning.

### Randomisation

The schools were allocated into intervention or control schools in five runs, one for each region. Before allocation each school was given three characteristics: Type (municipal or private), size (1–499 or 500 + pupils) and number of teachers included in the project (1 or 2–3). For each region, the second author received a list of the schools with an anonymous id concealing the true identity of the schools. The schools were first selected randomly and then allocated to intervention or control attempting to balance the three characteristics between the two groups. Finally, this anonymous allocation list with the anonymous id was linked to the true identity of the school, creating the final allocation list.

The characteristics of the schools divided by randomisation group are shown in Table 2.

**Table 2:** Characteristics of included schools in the SELFCARE project. A cluster randomised trial testing a school teacher training programme to deliver the “.b” programme in 110 schools covering schools in all five Danish regions

Characteristics	Intervention schools **n = 54**	Control schools **n = 56**
School type, municipal (%)	37 (69)	40 (71)
Total number of pupils, median (q1,q3)	430 (163,728)	490 (235,625)
Number of teachers included in the project (%)		
1 teacher	23 (43)	26 (46)
2 teachers	17 (31)	21 (38)
3 teachers	14 (26)	9 (16)
Number of teachers included in the project, in total	97	94

### Blinding

It was not possible to blind either intervention providers or study participants regarding intervention allocation.

### Data collection and management

We collect and store data using the Research Electronic Data Capture (REDCap) tool hosted by Aarhus University. REDCap is a secure, web-based application designed to support data capture for research [46]. The teachers will be asked to register the school class (class name, number of pupils, number of boys and girls), recruited for the project in RED-Cap. We inform the parents about the trial and the possibility to opt out of their child completing questionnaires in the trial via the school teachers. The teachers will register pupils with parental opt out in RED-Cap. The teachers will be asked to arrange time during school hours (in defined time-windows of two weeks) for the online completion of questionnaires and provide the pupils (including absentees) with the online access to the questionnaires. We e-mail two reminders to the teachers regarding the pupil questionnaires. A UNI-login is a unique identification number used in the Danish school system. It will be the key to link the three pupil questionnaires. The above procedure has been pilot tested and improved several times during the past years.

We will export data to a Stata file and data will be checked for double data entry and outliers.

### Statistical methods

Data will be analysed by a repeated measurement model with the systematic effect: region, type of school, school size, sex (pupil), class, time (three time points), intervention, interaction between time and intervention, and random effect of school, teacher/class within school and pupil within class.

Confidence intervals and standard errors will be found by bootstrapping to adjust for possible deviation from normality of the random effects.

We will perform four sensitivity analyses representing scenarios with data not missing at random. In these sensitivity analyses, missing outcomes will be substituted with predictions based on the analysis models above adding or subtracting 0.2*SD in the intervention or control arm.

### Trial status

Randomisation of the schools and teacher training have been completed. Recruitment, data collection and teaching of the “.b” programme among the pupils began in September 2020.

## Discussion

This trial will estimate the effectiveness of a population-based strategy on mental health in Danish schoolchildren. Mental health includes well-being and functioning. The benefits of mindfulness training are universally for the entire spectrum of mental health [16]. Mindfulness is a resource that has the potential to enhance the ability to regulate emotions, behavior, well-being, and social competences. Thus, mindfulness training may promote mental health and prevent poor mental health in schoolchildren. However, implementation challenges exist regarding how to train school teachers adequately in order to become competent to teach mindfulness in schools. Based on experiences from the UK and Denmark, we have developed a school teacher training programme to teach the evidence-based mindfulness school programme “.b” in Danish schools and will estimate the effectiveness of this programme as a part of compulsory class room teaching, both among a vulnerable subgroup and among the total population.

The trial´s strengths include a well powered, rigorously effectiveness study design; a cluster RCT with 3 and 6-month follow-up data, including municipal and private schools across Denmark. The latter will enhance the external validity of the trial. The results of the trial will be of great relevance for decision-makers of mental health promotion and prevention programmes in adolescents in European countries. It is a strength that the “.b” teaching is embedded in the compulsory class room teaching making it possible to reach an entire population of school children without causing stigma to anyone. We use the same manualized “.b”-programme and school teacher training programme, currently being evaluated in a large scale trial in the UK [16]. An additional strength includes addressing implementation issues discovered in the UK [24] and adds support for implementation in our school teacher training programme. This added third part of our school teacher training programme is based on rigorous work of The Danish Association for Promoting Life Wisdom in Children”[30]. The first part of our school teacher training programme also differs to some extent from the teacher training route in the UK. The Danish school teachers participate in the evidence-based MBSR course, which is more intensive in both duration of sessions and home practice than the mindfulness programme used in the UK context [24]. MBSR is taught by MBSR teachers, who also take care of the the subsequent parts of the school teacher training programme. MBSR is delivered in groups consisting solely of school teachers in the project.

It is also an advantage that the pupils will complete the questionnaires during school hours, which may prevent drop-out of pupils in the analyses. However, it is a limitation that we only use self-reported outcome measures, and cannot exclude the possibility of information problems. It may be a disadvantage that we evaluate the effectiveness of “.b” based on the school teachers´ second delivery of the “.b” programme, as they have only had the opportunity to become familiar with the material once before in “real-life”. It cannot be ruled out that the school teachers´ mindfulness teaching competences and the effectiveness of the “.b”-programme may improve with more teaching experience [24]. It is also a limitation that we have not yet planned a more rigorous implementation evaluation. However, we plan to conduct post-hoc interviews among participating pupils and teachers on their experiences with the training.

In conclusion, this trial will in a rigorous cluster RCT design, estimate the effectiveness of a population-based strategy on mental health in Danish schoolchildren. It evaluates the effect of a school teacher training programme to teach the “.b” programme that takes into account implementation issues. “.b” will be taught as a part of compulsory class room teaching. Effectiveness will be evaluated both in a vulnerable subgroup and among the total population. The results may guide decision-makers of mental health promotion and prevention programmes in adolescents in European countries.

## Data Availability

The dataset will be available from the corresponding author by reasonable request after publication of planned articles.

## Abbreviations

SDQ: Strengths and Difficulties Questionnaire
WHO: World Health Organization
MBI: Mindfulness-based intervention
MBSR: Mindfulness-Based Stress Reduction
MBCT: Mindfulness-Based Cognitive Therapy
FOFBOC: Feet On Floor Bum On Chair
MISP: Mindfulness In Schools Project
WEMWBS: Warwick-Edinburgh Mental Wellbeing Scale
BRS: Brief Resilience Scale
DSWQ: School connectedness and bullying items from the Danish student well-being questionnaire
HRQoL: Health-related quality of life
CAMM: Child-Adolescent Mindfulness Measure
REDCap: Research Electronic Data Capture
